# A Randomized Trial Evaluating Personalized Versus Guideline-Based Well Follow-Up Strategies for Patients with Early Breast Cancer: Feasibility Outcomes

**DOI:** 10.3390/curroncol33040224

**Published:** 2026-04-17

**Authors:** Ana-Alicia Beltran-Bless, Emma Himmelman, Alexander Kim, Gregory R. Pond, Parvaneh Fallah, Terry Ng, John Hilton, Marie-France Savard, Gail Larocque, Kelly-Anne Baines, Kendra Primeau, Deanna Saunders, Danielle Allard, Lisa Vandermeer, Mark Clemons

**Affiliations:** 1 Department of Medicine, Division of Medical Oncology, The Ottawa Hospital and University of Ottawa, 501 Smyth Road, Ottawa, ON K1H 8L6, Canada; 2 Cancer Therapeutics Program, Ottawa Hospital Research Institute, Ottawa, ON K1H 8L6, Canada; 3 Escarpment Cancer Research Institute, McMaster University, Hamilton, ON L8V 1C3, Canada; 4 Ottawa Hospital Cancer Centre, The Ottawa Hospital, Ottawa, ON K1H 1C4, Canada

**Keywords:** breast cancer, post-treatment, surveillance, follow-up

## Abstract

Despite the paucity of high-quality data supporting its benefits, routinely scheduled, in-person post-treatment surveillance of early breast cancer (EBC) patients remains common. Evaluation of different follow-up strategies is required. We present the feasibility phase of an ongoing randomized controlled trial (RCT) comparing two different follow-up strategies. Patients with EBC who completed the acute phase of their treatment were randomized to receive either personalized or ASCO guideline-based follow-up care alone. Feasibility endpoints, including rate of accrual, physician participation, patient acceptance of randomization arm, and patient retention 1 year after randomization, are presented. Of 279 patients approached, 261 (93.5%) were eligible and provided consent. Median rate of accrual was 34.5 patients per month, and all healthcare providers who agreed to study participation (*n* = 11) approached patients. Patients were randomized to receive personalized (*n* = 131) or guideline-based (*n* = 130) follow-up. No patients declined their randomization arm. For all 261 randomized patients, the 1-year participant retention rate was 92.0% (240/261). This RCT confirms both patient and healthcare-provider enthusiasm for studies comparing different strategies for post-treatment surveillance. No patients withdrew consent post-randomization due to a preference for one study arm over the other. While clinical effectiveness and patient satisfaction remain to be analyzed, our reported rates of attrition can be used by others when designing similar studies.

## Simple Summary

Routine in-person follow-up after early breast cancer treatment is common, but evidence for the best approach does not exist. We tested the feasibility of a trial comparing personalized follow-up to standard guideline-based care. Of the 279 patients approached, 261 agreed to participate, all accepted their assigned follow-up plan, and all participating healthcare providers actively enrolled patients. One-year retention was 92%, with no withdrawals due to group assignment. These results show that both patients and providers are willing to participate in studies testing different follow-up strategies. The findings provide practical guidance for designing future trials and support the feasibility of conducting larger studies to determine the most effective and patient-centered approach to post-treatment breast cancer care.

## 1. Introduction

Despite the paucity of high-quality data supporting its benefits, routinely scheduled, in-person post-treatment surveillance of patients with early breast cancer (EBC) remains common. The frequency of these visits varies between institutions and practice guidelines. For example, while the National Institute for Health and Care Excellence (NICE) in the UK does not recommend specific routine follow-up, both the American Society of Clinical Oncology (ASCO) and the European Society for Medical Oncology (ESMO) recommend regular follow-up with healthcare providers every 3 to 6 months for several years [1,2,3]. Even in practice, there is considerable variability in how these guidelines are implemented; for example, a real-world study in the Netherlands found that many low-risk patients received more frequent visits and imaging than their risk would suggest, demonstrating inconsistencies between risk and follow-up intensity [4].

While guidelines state that these routine visits may benefit patients via earlier detection of local and distant recurrences, management of the long-term effects of cancer and/or its treatment, patient reassurance, and the provision of advice regarding general health promotion, little evidence supports this [1,5,6,7,8]. Indeed, studies show that recurrences are rarely detected at routinely scheduled visits [9,10,11] and that neither patient nor healthcare-provider needs are being met by current strategies [9,12,13,14,15]. Evidence, however, does show that de-intensification of follow-up is safe and effective, and it reduces the economic burden of cancer care for both patients and the healthcare system, with no impact on disease-related outcomes [5,13].

Given the resource implications of different follow-up strategies and the global shift toward personalized medicine and survivorship models [16,17,18], there is a pressing need for randomized clinical trials (RCTs) to address how to optimize post-treatment surveillance [19]. There are, however, challenges with performing such studies, including the fact that using breast cancer survival endpoints requires a large number of patients and prolonged follow-up. For example, a theoretical RCT comparing the non-inferiority of ASCO guideline-based follow-up with de-escalated follow-up, using time to recurrence detection as the primary outcome, with a hazard ratio for recurrence of 1.2, would require a sample size of around 4654 patients to achieve 80% power [20]. Performing such a study would be cost-prohibitive, and therefore other clinically relevant endpoints would be needed. The choice of study endpoint also needs to reflect patient perceptions as to the importance of routine follow-up even in the absence of survival benefits, as well as the desire of HCPs for more individualized and risk-adapted post-treatment surveillance strategies [12,15].

Our group is currently performing an RCT comparing a more personalized follow-up strategy (ASCO guideline-based follow-up care, in addition to annual phone call post-mammogram plus on-demand telephone access to a nurse at our post-treatment surveillance program) with ASCO guideline-based follow-up alone. The patient-centered endpoints include patient quality of life (QoL), patient-reported fear of recurrence, anxiety, treatment-related toxicity, recurrences, and healthcare utilization [21]. Given the importance of studies in this setting, we have evaluated the feasibility of performing such trials by assessing endpoints such as rates of accrual, physician participation, patient acceptance of their randomization arm, and the number of patients remaining in the study 1 year after randomization.

Reporting these interim feasibility outcomes is critical in providing insight into the ability to conduct similar randomized trials in real-world clinical settings. Sharing these interim outcomes is important because they demonstrate that randomized trials comparing different post-treatment follow-up strategies are not only possible but also supported and embraced by both patients and healthcare providers. These findings, presented in the current manuscript, can serve as a practical guide for investigators planning similar trials in this field.

## 2. Materials and Methods

### 2.1. Study Population

Patients with early-stage breast cancer (stages I to III) who had completed the acute phase of their breast cancer treatment (i.e., surgery, chemotherapy/HER2-based therapy, and radiotherapy) and commenced endocrine therapy (if applicable) were eligible. At our center, these patients are referred by their medical or radiation oncologist to a dedicated Wellness Beyond Cancer Program (WBCP) for the coordination of routine, regularly scheduled follow-up with either the patient’s family practitioner team or a WBCP nurse practitioner [22]. These guidelines follow the ASCO guideline-based follow-up care recommendations, which include regular examinations with yearly mammographic evaluation, history and physical examination every 3 to 6 months for the first 3 years after primary therapy, every 6 to 12 months for the next two years, and then annually [2]. Routine blood tests, other imaging studies, and tumor marker testing are not recommended [2]. Exclusion criteria for referral to the WBCP program included patients who were receiving ongoing treatments with adjuvant agents such as zoledronate, luteinizing hormone-releasing hormone, abemaciclib, or olaparib. Patients with a previous history of invasive breast cancer, recurrent breast cancer, or metastatic breast cancer were also excluded.

### 2.2. Study Design

In this open-label, single-center study, patients were approached by a member of their circle of care during a routine clinic visit. Eligible patients who provided integrated informed oral consent [23] were randomized 1:1 to either a personalized follow-up strategy (ASCO guideline-based follow-up care, in addition to annual phone call post-mammogram plus on-demand telephone access to a nurse from the WBCP program) or to ASCO guideline-based follow-up care (current standard of care).

### 2.3. Intervention and Outcomes

Both personalized and guideline-based follow-up strategies adhered to ASCO guidelines. Patients randomized to receive personalized follow-up care were also telephoned by a nurse from the WBCP after the patient’s yearly mammogram and had on-demand telephone access to a nurse from the WBCP program. During the yearly phone call, patient concerns were addressed by a WBCP nurse or forwarded to their healthcare provider for further follow-up. Patients with bilateral mastectomies for whom mammographic evaluation was not performed were still contacted once per year. The primary outcome for the RCT, patient QoL, was measured using the validated FACT-G questionnaire [24,25]. Secondary outcomes included fear of recurrence, as determined via FACIT-FRQ questionnaire [26,27,28]; anxiety, as determined via HADS questionnaire [29]; and treatment-related toxicity concerns using the FACT-ES questionnaire [30]. These questionnaires were sent to patients at the time of randomization (baseline), and then 6, 12, and 24 months post-randomization. Data on the number and location of recurrences, as well as the frequency and type of healthcare contacts, were recorded in the follow-up questionnaires. Healthcare contacts were in the form of emergency room visits, planned/unplanned healthcare-provider visits, and phone calls/e-mails to the patient support line. Patient-reported healthcare utilization and recurrences were cross-referenced with their charts. Health utility values will be derived from the FACT-G questionnaire using the FACT-8D algorithm, and health economic evaluation results will be presented as cost per QALY gained upon completion of the study [25].

Study questionnaires were integrated into an electronic database developed by the Ottawa Methods Centre. Participants who opted to receive electronic copies of the questionnaires were provided with a secure link leading to the REaCT Patient Portal, where they were able to access and complete them. Paper questionnaires were sent to participants, if that was their preference, or to those without access to a device with internet connection. Participants were also able to complete questionnaires via phone call.

While data collection for the primary and secondary endpoints is ongoing, given the importance of studies in this setting, we also want to evaluate the feasibility of performing such trials. This would be assessed using endpoints such as rates of accrual, physician participation, patient acceptance of their randomization arm, and the number of patients remaining in the study 1 year after randomization.

### 2.4. Statistical Methods

Sample size for the RCT was calculated based on a clinically important difference (CID) described by Cella et al. to be an observed change in FACT-G of 6.8 points at 2 months [31]. The personalized follow-up arm will be of interest for further study if patient quality of life is not worse than standard-of-care follow-up; hence, a non-inferiority design will be used, and the non-inferiority margin will be set at ¾ of the CID (or 5.1 points). It is also assumed that the standard deviation of the change score is 15 points (approximated based on Tables 4 and 5 of Cella et al.) [31]. Using a one-sided α = 0.05 non-inferiority design, the study will have 80% power with 216 patients. To account for 5% loss to follow-up, target accrual was set to 228 patients. Descriptive statistics were used to summarize patient outcomes by intervention arm and were used to summarize baseline data.

## 3. Results

### 3.1. Baseline Patient Characteristics

**Figure A1:**
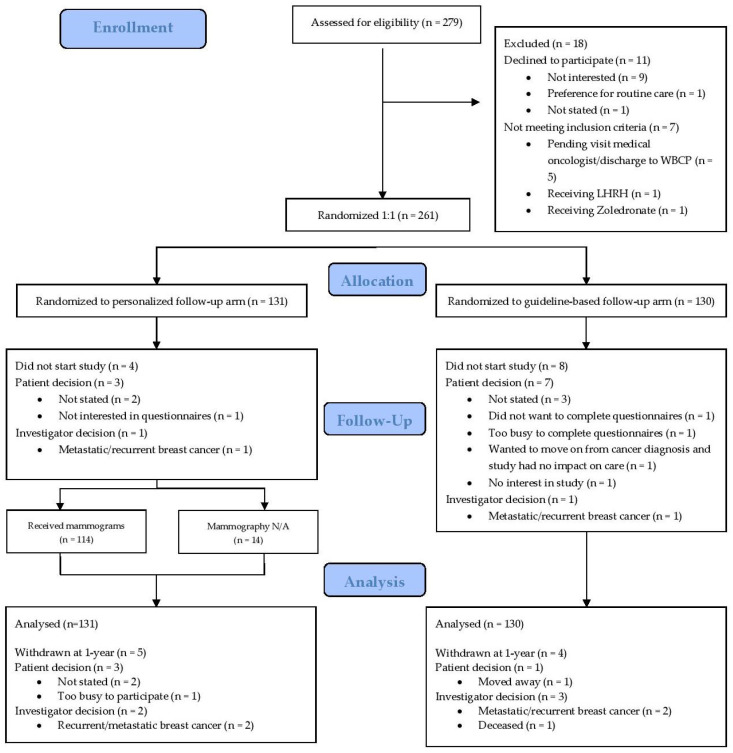
Consort diagram.

Between 19 August 2022 and 10 March 2023, 279 patients were approached for the study. Eleven of the 279 approached patients declined to participate in the study due to lack of interest in the study (*n* = 9), preference for routine care (*n* = 1), and reason unstated (*n* = 1). Of the 279 patients approached, 261 (93.5%) were eligible, provided informed integrated oral consent, and randomized 1:1 to receive personalized follow-up care (*n* = 131) or follow-up care adhering to ASCO guideline-based follow-up care (*n* = 130). The CONSORT diagram is shown in Figure A1. The baseline characteristics of 258 female and 3 male patients are shown in Table 1. The median age of all randomized patients was 63 years (IQR = 16 years). Most patients were postmenopausal, accounting for 73.2% (*n* = 191/258) of all female patients. Most patients, 84.37% (*n* = 220/261), had ER/PR-positive disease, 16.5% (*n* = 43/261) had HER2-positive disease, and 15.3% (40/261) had triple-negative disease. While 115 (44.1%) had received prior chemotherapy, 200 (76.6%) were receiving endocrine therapy. The median time from surgery to study randomization was 14 months (IQR 16.2) and 16 months (IQR 22.75 months) for patients treated with neoadjuvant and adjuvant therapy. A full breakdown of tumor characteristics is shown in Table A1.

### 3.2. Rate of Participant Accrual

**Figure A2:**
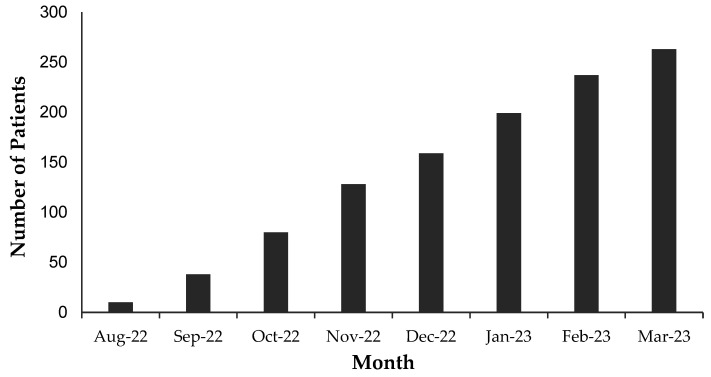
Patient accrual plot (cumulative).

Between 19 August 2022 and 10 March 2023, the median rate of accrual was 34.5 patients per month (IQR = 12.5). The fewest number of patients was accrued at the beginning of the study in August 2022 (10 patients per month), while the greatest number of patients was accrued in November 2022 (48 patients per month) (Figure A2).

### 3.3. Physician Participation

Patients were approached by all 11 participating physicians. Median accrual per physician was 12 (IQR = 15), ranging from 1 patient to 144 patients.

### 3.4. Patient Acceptance of Randomization Arm

Of the 261 eligible, consenting, and randomized patients, none requested to be withdrawn from the study due to dissatisfaction with their allocated randomization arm. One approached patient declined to participate prior to randomization in the study due to a preference for routine care.

### 3.5. Number of Patients Remaining in Study 1 Year Post-Randomization

The participant retention rate after a year post-randomization was 92.0% (240/261). Most withdrawals were patient-requested. While some patients were withdrawn for unstated reasons (*n* = 7), known reasons for patient-requested withdrawals included not wanting to complete study questionnaires (*n* = 2); wanting to move on from their cancer diagnosis (*n* = 2); moving away (*n* = 1); questionnaires being upsetting to complete (*n* = 1); and being too busy to participate in the study (*n* = 1). Seven patients were taken off the study by investigators due to development of metastatic breast cancer (*n* = 4), local recurrence (*n* = 1), contralateral breast cancer (*n* = 1), and death unrelated to breast cancer (*n* = 1) (Table 2).

## 4. Discussion

Despite the paucity of high-quality data supporting its benefits, routinely scheduled, in-person post-treatment surveillance of patients with EBC remains common. While the frequency of these visits varies between evidence-based guidelines [1,2,3,5,6], current strategies are not meeting the goals of either patients or healthcare providers [9,10,11,12,13,14]. For example, Artor et al. found that the majority of breast cancer recurrences were detected outside of scheduled surveillance visits, primarily through symptom-driven detection, highlighting that fixed-interval follow-up may not be the most effective strategy [32]. Similarly, this echoes results of our own study showing that routinely scheduled visits detect very few curable recurrences, further emphasizing the need for trials evaluating alternative surveillance strategies [9].

Current research is largely focused on advancing imaging techniques and methods for detecting distant recurrences, rather than questioning whether routine follow-up visits for asymptomatic early-stage breast cancer patients are necessary or how surveillance schedules could be optimized to better meet patient and healthcare-provider needs [4]. More high-quality data on optimal breast cancer surveillance are needed for several reasons, including the rising number of patients due to the increased incidence of breast cancer and treatment advances, the increasing demands on cancer specialists, decreasing patient access to primary care physicians, and the significant economic burden of survivorship care [16,19]. Consequently, the demand for post-treatment surveillance will continue to rise. This underscores the critical need for further clinical trials to optimize long-term patient management. While there is a growing body of evidence suggesting that de-intensification of follow-up is safe, effective, and reduces the economic burden of cancer care for both patients and the healthcare system, with no impact on disease-related outcomes [5,13], more studies are needed. As there is an increasing move toward more personalized care, the current study was designed to compare two different follow-up strategies, as well as the feasibility of performing clinical trials in this setting. We are sharing feasibility outcomes, such as patient accrual, physician participation, and acceptance of randomization, to highlight the strong interest, engagement, and willingness of both patients and healthcare providers to participate in such studies.

Within the setting of this pragmatic study, the rates of patient accrual, acceptance of randomization arm, physician participation, and 1-year retention rates were all high, demonstrating that studies evaluating different post-treatment surveillance strategies can be performed. A 1-year retention rate of 92% compares favorably with published longitudinal and cancer survivorship trials, where retention rates of approximately 80–90% are typical, and ≥80% is often considered indicative of high retention [33,34]. Patient acceptance of their randomization arm was high, and notably, no randomized patients requested to be withdrawn due to their randomization arm. Additionally, all participating physicians approached and enrolled patients for the study. As with all clinical trials, attrition was present. Our original study design included a 5% loss-to-follow-up rate. By one year post-randomization, the participant retention rate was 92.0% (240/261). As the trial is following patients for 2 years post-randomization, additional patient attrition is to be expected, and therefore, future trial designs will need to take this into account as part of their design.

As with all trials, the current study has limitations. First, it was a single-center study using a primary endpoint not based on breast cancer survival outcomes. However, the pragmatic design of the study, including the use of oral integrated consent, increases the accessibility of clinical trials to all patients and allows study populations to better reflect those observed in real-world practice. In addition, while our study team has an interest in comparing de-intensification post-treatment surveillance studies, we firstly wanted to evaluate patient and healthcare-provider interest in entering such trials. The current study was not a de-escalation study, as patients were effectively randomized to either standard-of-care post-treatment surveillance or standard of care plus more care (i.e., the post-mammogram phone call plus on-demand telephone access to a nurse from the WBCP program). Whether a study with an arm with less than “standard of care” would have accrued rapidly remains to be studied.

By reporting these interim feasibility outcomes, we aim to demonstrate that both patients and healthcare providers are willing and motivated to participate in trials evaluating post-treatment follow-up strategies, an area where interest and high-quality evidence have historically been limited. Understanding rates of patient accrual, provider engagement, acceptance of randomization, and retention helps identify potential challenges and informs strategies to improve recruitment and adherence in future studies. These data are also critical for planning adequately powered trials, estimating sample sizes, and ensuring that subsequent studies assessing clinical effectiveness and patient-centered outcomes can be successfully completed with minimal attrition or bias, ultimately supporting the generation of evidence to optimize follow-up care for breast cancer survivors.

While the current manuscript demonstrates the feasibility of our proposed design, there is a clear need for future studies to evaluate different endpoints, such as recurrence outcomes, and to explore different surveillance strategies, including de-intensification of surveillance. Given the rarity of detecting asymptomatic disease recurrence during routine appointments, future trials using disease recurrent outcomes as a primary outcome will require large and possibly prohibitive sample sizes [20]. Irrespective of funding and sample size issues, further studies are necessary to evaluate alternatives to the current “one-size-fits-all” approach endorsed by many guidelines. Current strategies do not take into account individual expectations of post-treatment surveillance [16,17], the individual patient’s risk of recurrence, or the fact that routine clinical examination rarely detects recurrence at a more “curable” stage [9,11].

One group has developed a tool called INFLUENCE, which was designed to calculate local recurrence risk and contralateral breast primary risk to support physicians in personalizing scheduling of surveillance visits [18]. In addition, future studies will need to incorporate the findings of the MAMMO-50 trial, which showed, for patients aged 50 years or older and at 3 years post-diagnosis, less frequent mammograms were non-inferior compared with annual mammograms for breast cancer-specific survival, recurrence-free interval, and overall survival, and should be considered for this population [35].

## 5. Conclusions

Despite the paucity of high-quality data supporting its benefits, routinely scheduled, in-person post-treatment surveillance of patients with EBC remains common. RCTs comparing alternate strategies are needed. The current study confirms the feasibility of such studies, but the rates of attrition mean that both the study sample size and the enrollment period should be accounted for when designing such studies.

## Figures and Tables

**Table 1:** Baseline characteristics of study participants.

	A: Personalized Follow-Up (*n* = 131)	B: Guideline-Based Follow-Up (*n* = 130)	Total (*n* = 261)
Characteristics	*n* (%)	*n* (%)	*n* (%)
**PATIENT CHARACTERISTICS** Sex			
Female	130 (99.2)	128 (98.5)	258 (98.9)
Male	1 (0.8)	2 (1.5)	3 (1.1)
Age (years)			
Median (IQR)	62 (54–71)	65 (55–72)	63 (55–71)
Menopausal Status			
Pre-	25 (19.1)	25 (19.2)	50 (19.2)
Peri-	10 (7.6)	7 (5.4)	17 (6.5)
Post-	95 (72.5)	96 (73.9)	191 (73.2)
Unknown	0 (0.0)	0 (0.0)	0 (0.0)
N/A (Male)	1 (0.8)	2 (1.5)	3 (1.1)
Initial Tumor Detection			
Screen-detected	64 (48.9)	69 (53.1)	133 (51.0)
Symptomatic	65 (49.6)	60 (46.1)	125 (47.9)
Unknown	2 (1.5)	1 (0.8)	3 (1.1)
**TUMOR CHARACTERISTICS** Histologic Subtype			
Classic lobular	15 (11.5)	12 (9.2)	27 (10.3)
Ductal and NOS	98 (74.8)	97 (74.6)	195 (74.7)
Other	18 (13.7)	21 (16.1)	39 (15.0)
Initial Stage (either pathology-based for adjuvant patients or clinically for neoadjuvant patients)			
I	81 (61.8)	83 (63.9)	164 (62.8)
II	37 (28.2)	36 (27.7)	73 (28.0)
III	13 (9.9)	11 (7.7)	24 (9.2)
Unstaged	0 (0.0)	0 (0.0)	0 (0.0)
Grade			
1	23 (17.6)	20 (15.4)	43 (16.5)
2	70 (53.4)	66 (50.8)	136 (52.1)
3	38 (29.0)	42 (32.3)	80 (30.6)
Ungraded	0 (0.0)	2 (1.5)	2 (0.8)
ER/PR Expression			
Positive	114 (87.0)	106 (81.5)	220 (84.3)
Negative	17 (13.0)	23 (17.7)	40 (15.3)
Unknown ^†^	0 (0.0)	1 (0.8)	1 (0.4)
HER2 Expression			
Positive	23 (17.6)	20 (15.4)	43 (16.5)
Negative	108 (82.4)	107 (82.3)	215 (82.3)
Unknown	0 (0.0)	3 (2.3)	3 (1.2)
**TREATMENT CHARACTERISTICS** Type of breast surgery			
Mastectomy	46 (35.1)	36 (27.7)	82 (31.4)
Lumpectomy	85 (64.9)	94 (72.3)	179 (68.6)
Type of axillary lymph node surgery ^‡^			
SLNB	116 (88.5)	118 (90.8)	234 (89.7)
ALND	12 (9.2)	9 (6.9)	21 (8.0)
Not done (Nx)	3 (2.3)	3 (2.3)	6 (2.3)
Neoadjuvant therapy			
Yes	20 (15.3)	21 (16.2)	41 (15.7)
Endocrine therapy			
Yes	102 (77.9)	98 (75.4)	200 (76.6)
Chemotherapy			
Yes	56 (42.7)	59 (45.4)	115 (44.1)
Radiotherapy			
Yes	103 (78.6)	102 (78.5)	205 (78.5)
TIME FROM DEFINITIVE BREAST SURGERY TO RANDOMIZATION			
Median (IQR) (months)	15 (10–32)	15 (9–31)	15 (9–31)

Abbreviations: ER, estrogen receptor; PR, progesterone receptor; HER2, human epidermal growth factor receptor 2; NOS, not otherwise specified; SLNB, sentinel lymph node biopsy; ALND, axillary lymph node dissection. ^†^ Instances where both ER and PR expression were unknown. ^‡^ Few patients have received both SLNB and ALND.

**Table 2:** Reasons for study withdrawal within 1 year post-randomization.

	A: Personalized Follow-Up (*n* = 9)	B: Guideline-Based Follow-Up (*n* = 12)
Reasons for Withdrawal	*n* (%)	*n* (%)
Patient-requested (*n* = 14)		
Not stated	3 (50.0)	4 (50.0)
Did not want to complete questionnaires	1 (16.7)	1 (12.5)
Wanted to move on from cancer diagnosis	1 (16.7)	1 (12.5)
Too busy to participate	1 (16.7)	0 (0.0)
Moved away	0 (0.0)	1 (12.5)
Questionnaires were upsetting to complete	0 (0.0)	1 (12.5)
Investigator decision (*n* = 7)		
Recurrent/metastatic breast cancer	3 (100)	3 (75.0)
Deceased	0 (0.0)	1 (25.0)

## Data Availability

The data presented in this study are available from the corresponding author upon request due to restrictions imposed by institutional research ethics approval and patient confidentiality considerations. Please direct requests to the senior author at mclemons@toh.ca.

## Appendix A

**Table A1:** Baseline clinical and pathological TN categories of patients who received neoadjuvant therapy and pathological TN categories of patients who received surgery as initial treatment.

	A: On-Demand Personalized Follow-Up	B: Guideline-Based Follow-Up	Total
	*n* (%)	*n* (%)	*n* (%)
Neoadjuvant Patients—Baseline cT Category (*n* = 43)			
T1mi	0 (0.0)	0 (0.0)	0 (0.0)
T1a	0 (0.0)	0 (0.0)	0 (0.0)
T1b	1 (4.5)	0 (0.0)	1 (2.3)
T1c	2 (9.1)	0 (0.0)	2 (4.6)
T2	12 (54.5)	13 (61.9)	25 (58.1)
T3	5 (22.7)	7 (33.3)	12 (27.9)
T4a	1 (4.5)	1 (4.8)	2 (4.6)
T4b	0 (0.0)	0 (0.0)	0 (0.0)
T4c	0 (0.0)	0 (0.0)	0 (0.0)
T4d	1 (4.5)	0 (0.0)	1 (2.3)
Neoadjuvant Patients—Baseline cN Category (*n* = 43)			
N0	12 (54.5)	12 (57.1)	24 (55.8)
N1	5 (22.7)	7 (33.3)	12 (27.9)
N1mi	1 (4.5)	0 (0.0)	1 (2.3)
N2a	2 (9.1)	1 (4.8)	3 (7.0)
N2b	0 (0.0)	0 (0.0)	0 (0.0)
N3a	1 (4.5)	1 (4.8)	2 (4.6)
N3b	0 (0.0)	0 (0.0)	0 (0.0)
N3c	0 (0.0)	0 (0.0)	0 (0.0)
Unknown	1 (4.5)	0 (0.0)	1 (2.3)
Neoadjuvant Patients—Baseline pT Category (*n* = 43)			
T0	6 (27.3)	6 (28.6)	12 (27.9)
Tis	2 (9.1)	1 (4.8)	3 (7.0)
T1mi	1 (4.5)	0 (0.0)	1 (2.3)
T1a	0 (0.0)	4 (19.0)	4 (9.3)
T1b	1 (4.5)	3 (14.3)	4 (9.3)
T1c	5 (22.7)	3 (14.3)	8 (18.6)
T2	6 (27.3)	4 (19.0)	10 (23.3)
T3	1 (4.5)	0 (0.0)	1 (2.3)
T4a	0 (0.0)	0 (0.0)	0 (0.0)
T4b	0 (0.0)	0 (0.0)	0 (0.0)
T4c	0 (0.0)	0 (0.0)	0 (0.0)
T4d	0 (0.0)	0 (0.0)	0 (0.0)
Neoadjuvant Patients—Baseline pN Category (*n* = 43)			
N0	13 (59.1)	14 (66.7)	27 (62.8)
N1	2 (9.1)	4 (19.0)	6 (14.0)
N1mi	4 (18.2)	2 (9.5)	6 (14.0)
N2a	1 (4.5)	1 (4.8)	2 (4.6)
N2b	0 (0.0)	0 (0.0)	0 (0.0)
N3a	1 (4.5)	0 (0.0)	1 (2.3)
N3b	0 (0.0)	0 (0.0)	0 (0.0)
N3c	0 (0.0)	0 (0.0)	0 (0.0)
Nx	1 (4.5)	0 (0.0)	1 (2.3)
Patients w/Surgery as Initial Treatment—Baseline pT Category (*n* = 218)			
T0	0 (0.0)	0 (0.0)	0 (0.0)
Tis	0 (0.0)	0 (0.0)	0 (0.0)
T1mi	0 (0.0)	3 (2.8)	3 (1.4)
T1a	5 (4.6)	7 (6.4)	12 (5.5)
T1b	10 (9.2)	12 (11.0)	22 (10.1)
T1c	46 (42.2)	51 (46.8)	97 (44.5)
T2	39 (35.8)	31 (28.4)	70 (32.1)
T3	9 (8.3)	5 (4.6)	14 (6.4)
T4a	0 (0.0)	0 (0.0)	0 (0.0)
T4b	0 (0.0)	0 (0.0)	0 (0.0)
T4c	0 (0.0)	0 (0.0)	0 (0.0)
T4d	0 (0.0)	0 (0.0)	0 (0.0)
Unknown	0 (0.0)	0 (0.0)	0 (0.0)
Patients w/Surgery as Initial Treatment—Baseline pN Category (*n* = 218)			
N0	67 (61.5)	73 (67.0)	140 (64.2)
N1	27 (24.8)	19 (17.4)	46 (21.1)
N1mi	11 (10.1)	11 (10.1)	22 (10.1)
N2a	1 (0.9)	0 (0.0)	1 (0.5)
N2b	0 (0.0)	0 (0.0)	0 (0.0)
N3a	1 (0.9)	3 (2.8)	4 (1.8)
N3b	0 (0.0)	0 (0.0)	0 (0.0)
N3c	0 (0.0)	0 (0.0)	0 (0.0)
Nx	2 (1.8)	3 (2.8)	5 (2.3)
Unknown	0 (0.0)	0 (0.0)	0 (0.0)
